# Fluid Overload

**DOI:** 10.3389/fvets.2021.668688

**Published:** 2021-06-29

**Authors:** Bernie Hansen

**Affiliations:** Department of Clinical Sciences, College of Veterinary Medicine, North Carolina State University, Raleigh, NC, United States

**Keywords:** fluid overload, hypervolemia, edema, effusion, fluid balance, resuscitation, stabilization

## Abstract

Fluid overload (FO) is characterized by hypervolemia, edema, or both. In clinical practice it is usually suspected when a patient shows evidence of pulmonary edema, peripheral edema, or body cavity effusion. FO may be a consequence of spontaneous disease, or may be a complication of intravenous fluid therapy. Most clinical studies of the association of FO with fluid therapy and risk of harm define it in terms of an increase in body weight of at least 5–10%, or a positive fluid balance of the same magnitude when fluid intake and urine output are measured. Numerous observational clinical studies in humans have demonstrated an association between FO, adverse events, and mortality, as have two retrospective observational studies in dogs and cats. The risk of FO may be minimized by limiting resuscitation fluid to the smallest amount needed to optimize cardiac output and then limiting maintenance fluid to the amount needed to replace ongoing normal and pathological losses of water and sodium.

The mammalian stress response to injury, hypovolemia, or critical illness includes retention of sodium and water and, at least early on, increased thirst (1–5). These responses may serve to defend blood volume and maintain hydration when access to water is impaired by debility, and in the absence of medical care likely confer some survival advantage. However, the application of modern intensive care sets the stage for harm when potentially limitless amounts water and sodium can be administered to patients whose upset biology favors retention of both. Although evolutionary pressure likely selected for the adaptive responses to hypovolemia following injury or illness, there was no such selection pressure to respond to hypervolemia in the same setting, a situation now commonly referred to as fluid overload (FO).

The concept of FO as a clinical entity to be avoided appeared in earnest within the medical literature during the 1970's, with 54 PubMed citations from that decade using that phrase. Although for many years FO has been recognized as a potential complication of anuria in chronic hemodialysis patients, in this century the phrase has more often been used to describe a complication of fluid therapy in any patient at risk for hypervolemia or edema. The importance of FO has been underlined by a growing number of reports of observational studies that associate the condition with higher morbidity and mortality in hospitalized patients, an association that held true in recent meta-analyses of 44 studies in children (6) and 31 in adults (7).

## Causes of FO

Clinically, FO is usually defined by some combination of edema, excessive weight gain, or excessively positive fluid balance in a patient that has received intravenous fluid therapy. In fact, FO is almost universally defined in such terms in clinical studies of humans (7, 8). However, some have argued that rather than focusing on development of edema as a foundational feature of fluid overload, clinicians should be more concerned about the presence of hypervolemia (9). Hypervolemia is a state of excessive blood volume and elevated mean circulatory filling pressure (MCFP). Mean circulatory filling pressure is in turn defined as the average transmural pressure of the circulatory system when the heart and blood flow is stopped, and it is determined by blood volume and autonomic control of vascular smooth muscle. Its value is typically close to the average transmural pressure at the level of systemic post-capillary venules, and the pressure gradient between those venules and the right atrium (central venous pressure) is the driving force returning blood to the heart (10). In animals with a normally functioning heart, intravenous fluid therapy increases cardiac output primarily by increasing the pressure difference between the MCFP and the central venous pressure.

If the MCFP becomes sufficiently elevated by circulatory failure or fluid overload, the elevated venule pressure requires higher capillary pressures to maintain blood flow, and increased capillary pressure promotes fluid movement to the interstitial space. A high rate of fluid administration alone is not enough to produce FO (at least when within a clinically relevant range); development and maintenance of FO requires impaired excretion of water or abnormal function of the interstitial compartment, or both. For example, administration of 90 mL/kg of lactated Ringer's solution in 1 h to mildly dehydrated dogs produces none of the features of FO, and some features of FO seen during administration of 360 mL/kg in 1 h largely resolve within 30 min, accompanied by voiding large quantities of urine (11).

Hypervolemia secondary to impaired excretion of excess fluid may be seen in animals with heart, kidney, or liver disease, and some animals with these chronic conditions present for care precisely because they have clinical signs of FO. Organ dysfunction as a component of acute illness—for example, impaired heart and kidney function observed in some dogs with septic shock—may contribute to FO in the face of overzealous replacement fluid administration. Another factor contributing to impaired excretion of excess water is dissociation of arginine vasopressin (AVP) release from osmoregulation, a situation brought about by disorders and drugs that result in water retention, hyponatremia, and edema. As reviewed by Moritz and Ayus, the list of conditions associated with hospital-acquired hyponatremia due to excessive secretion of AVP and water retention is quite long and varied (12). Water retention is a particularly important issue during treatment of hospitalized children with hypotonic maintenance fluids, who often receive an excess of water via the commonly used Holliday-Seger formula^1^ (13). Although FO is an occasional complication, neurological consequences of hyponatremia are the more serious side effects of excessive administration of hypotonic fluid to patients prone to water retention. Stimuli for excessive AVP release during inflammatory states includes an increased plasma concentration of interleukin-6, particularly during sepsis and tissue injury from trauma. Local production of interleukin-6 in response to osmotic stimuli is a physiological stimulus of hypothalamic AVP production, and elevated plasma concentrations secondary to systemic illness (sepsis in particular) appears to have similar effects on AVP production and release, independent of osmoregulation (14).

As anyone who has examined a patient with a soft tissue infection will recognize, inflammation also promotes edema in ways that are independent of volume status and AVP release. As described by Bhave and Neilson inflammatory states yield a reduction in interstitial fluid pressure by disrupting the tension of the collagen fibrils responsible for maintaining a tight interstitial matrix, increasing the compliance of the interstitial compartment (15). Under physiological conditions, interstitial fluid pressure is under local control via cellular connections to the collagen matrix through integrin receptors that are in turn linked to the cellular actin cytoskeleton. Inflammation may cause depolymerization of the cytoskeleton and break the integrin links to collagen, loosening the matrix and causing interstitial fluid pressure to fall, favoring fluid movement from the capillary.

Regardless of whether edema was initially caused by an increased MCFP or reduced interstitial pressure, once interstitial fluid volume has grown enough to increase tissue weight by more than 10–20% the compartment becomes highly compliant (16). This increase in compliance allows the interstitial compartment to accommodate large quantities of additional fluid without much of an increase in pressure, serving to maintain edema once it has begun.

In the case of edema caused by intravenous fluids administered to animals with systemic inflammation, there are also contributions from reduced plasma albumin concentration and disruption of the endothelial barrier to albumin. The dilutional effect of fluid therapy and the acute phase response to reduce plasma albumin concentration decreases plasma oncotic pressure and favors fluid filtration. The capillary glycocalyx barrier to albumin may be compromised by inflammation (17), release of atrial natriuretic peptide secondary to hypervolemia (18), and rapid fluid administration (even in the absence of increased atrial natriuretic peptide) (19). Thus, both underlying disease and the fluid resuscitation to support the circulation can favor development of edema.

## Why is FO Harmful?

FO causes harm due to the effects of edema fluid in the interstitial space. In the lung, the presence of excess extravascular water impairs gas exchange, reduces pulmonary compliance, and increases the work of breathing, complications that reduce the oxygen content of blood and increase the amount of oxygen consumed by the muscles of ventilation. In the systemic circulation, FO may impair diffusion of oxygen and energy substrates, obstruct capillary blood flow and lymphatic drainage, distort tissue architecture, and impair cell-to-cell interactions. Every major organ system may manifest complications of the syndrome (Table 1), but the lungs and organs confined by rigid structures (brain) or capsules (kidney, liver) may be particularly vulnerable. It is important to recognize that by the time edema can be seen and felt at the body surface it is also occurring internally. Whereas, the skin can survive prolonged periods of reduced oxygen delivery and can maintain some of its barrier function despite distorted architecture, internal organs with higher basal oxygen consumption and more complex functional architecture cannot.

**Table 1:** Adverse effects of FO on organ function.

**Organ system**	**Potential complications**	**Examples of evidence**
Brain	•Cognitive impairment •Delirium •Increased ICP/decreased CPP	•Mechanically ventilated patients with FO have longer periods of delirium/coma after extubation (20) •Cortical necrosis observed in a dog with FO (21)
Gastrointestinal tract	•Ileus •Increased permeability to bacterial translocation •Impaired liver function •Increased intra-abdominal pressure/!!breakcompartment syndrome	•Prolonged ileus is associated with FO in humans (22) •Higher wound infection rates associated with FO (23) •Strong association between resuscitation fluid and compartment syndrome (24)
Heart	•Myocardial edema •Arrhythmia •Impaired systolic and diastolic function	•Elevated troponin and pressor requirements associated with fluid resuscitation (25)
Kidneys	•Increased interstitial pressure •Decreased RBF/GFR •AKI	•Association of FO with AKI (26)
Lungs	•Pulmonary edema •Pleural effusion	•Improved lung function in patients treated with conservative fluid therapy (27)
Skin/muscle	•Edema •Weakness	•Delayed healing of abdominal wound closure (28)

## Diagnosis

Although FO is often first diagnosed based on recognition of edema or effusion, a better approach is to identify it earlier by monitoring changes in body weight or cumulative fluid balance. Measuring change in body weight has been considered the gold standard clinical measurement approach to monitor fluid balance in hospitalized humans since the 1970's and has been used to monitor critically ill companion animals (29, 30). However, because of the difficulty in obtaining and charting accurate weights in critically ill humans, a more common strategy is to monitor “ins and outs,” that is, cumulative fluid administration vs. the sum of cumulative urine production, drainage losses, and sometimes volume estimates of diarrhea and insensible losses. This approach requires an indwelling urinary catheter or other means to accurately quantify urine production. Fluid balance is often used as a surrogate for changes in weight, but some studies in adults (31, 32) and neonates (33) have demonstrated extremely poor correlation between the two measurements. Technical reasons for the discrepancy include charting errors in recording fluid balance and inaccurate/erratic techniques used to obtain multiple weight measurements. One small study of 32 human cardiac surgery patients comparing FO to WG calculations identified 25 arithmetic errors in nursing charts, a finding that emphasizes the potential for caregiver error to interfere with even objective patient assessments (32). Other potential reasons for discrepancies include failure to accurately measure gastrointestinal losses or loss from wound or body cavity drainage, inaccurate prediction of insensible water losses, and the unpredictable rate of catabolic loss of tissue.

The advent of more widespread use of intensive care beds with built-in scales may make routine use of weight change more practical for human patients. Veterinary application of weight change assessment in critically ill patients is impeded by several factors, including the expense of purchasing and maintaining the calibration of accurate scales, the need to lift patients onto scales, and the weight effects of monitoring devices, bandages, and bladder size in patients that often weigh less than human infants. Commonly used formulas for % weight gain and % fluid overload include these:

$$\begin{array}{l} \text{Weight gain }\left(\%\right)\\ =\frac{\text{Current }\left(\text{or maximal}\right)\text{ body weight }-\text{ baseline body weight}}{\text{baseline body weight}}\times \;100\\ \text{Fluid overload }\left(\%\right)=\frac{\text{Fluid intake }-\text{ fluid output}}{\text{baseline body weight}}\times \;100 \end{array}$$

The data used to populate these formulas may reflect the entire duration of hospitalization or may be used to monitor daily changes. In patients judged to be dehydrated at baseline, the formula may be modified to subtract the % dehydration (expressed in weight or volume of fluid, depending on the formula) from the numerator (34). Although there is not universal agreement on what degree of weight gain or FO represents a clinically important change, widely cited figures include 5 and 10%, with 10% marking a threshold for intervention.

The development of complications of FO probably depends not only on weight change or fluid balance, but also on the *distribution* of excess water. In critical illness there may be a variable relationship between total body water content and the compartmental distribution of water between extravascular and intravascular, and within the vascular compartment the distribution between the unstressed volume and the stressed volume that creates cardiac preload. An excess of extracellular water is more likely to produce clinical complications of FO than an excess of intracellular water. Supporting this is the observation of Bihari et al. that sodium balance—a key determinate of extracellular fluid volume—correlates better with respiratory dysfunction than does fluid balance (35).

The presence of hypervolemia has been assessed by physical exam findings, invasive hemodynamic pressure measurements, and ultrasound assessment. As reviewed by Beaubien-Souligny et al. (36), ultrasound techniques may identify hypervolemia in humans by documenting internal jugular vein distension, dilation of the inferior vena cava, reversal of systolic:diastolic hepatic vein flow, development of pulsatile portal vein flow, and discontinuous intrarenal venous flow. Ultrasound manifestations of increased intracranial pressure include an increase in the diameter of the optic nerve and a reduction in diastolic flow of the middle cerebral artery. The presence of increased extravascular lung water is revealed by an increase in the number of B lines.

Ultrasound examination is also used to identify patients that are likely to respond to intravenous fluids with an increase in cardiac output. Veterinary studies using ultrasound to predict fluid responsiveness have included assessment of caudal vena cava collapsability (37, 38) and prospective comparison of the CVC diameter to that of the abdominal aorta (38), where “fluid responsiveness” was defined as a >15% increase in ascending aorta velocity time index immediately following administration of a fluid bolus. Although the authors of a retrospective case series (37) concluded that the effect of administration of 30 mL/kg of LRS (at an unspecified rate) could be predicted by the magnitude of the respiratory effect on CVC diameter, a prospective study demonstrated that respiratory variation in CVC diameter did not predict the response to rapid (1 min) administration of 4 mL/kg of Hartmann's solution. In contrast, a ratio of the maximum CVC diameter to aortic diameter at the level of the porta hepatis of 0.83 had a sensitivity of 100% and a specificity of 75% for fluid responsiveness.

## FO as an Entity in Veterinary Patients

The author is aware of just three reports of small studies that addressed the incidence and impact of FO in dogs and cats. A retrospective study defined FO as development of symptomatic pulmonary edema or pleural effusion in 11 cats with urethral obstruction that were treated with intravenous fluids, and compared recorded data from those cats with 51 control cats that had urethral obstruction but did not develop respiratory signs (39). A “FO score” was calculated based on the % fluid overload formula (see above), and weight change between admission and the date that respiratory signs developed was calculated. Although the relationship between FO score and weight change was not described, some cats in both groups had negative fluid balance and some affected cats lost weight by the time they developed respiratory signs. Although the range of FO scores in the control cats was greater than that of the cats with FO, and included subjects with more severely negative and positive fluid balance, the median FO score in affected cats was significantly greater than the controls (6 vs. 2.46%). Ten affected cats developed a cardiac gallop, and echocardiography identified underlying heart disease in 5 of 6 cats examined; therefore, occult heart disease was likely the single most important factor in the development of clinical signs.

Another veterinary report defined FO as a positive fluid balance in dogs (after correcting dehydration) that were monitored with a closed urine collection system, and compared outcomes between 34 dogs with critical illness and 15 hemodynamically stable dogs with neuro-orthopedic disease that had closed urine collection systems in place to assist with nursing care (34). Fluid balance and % FO were examined as continuous data, and correlation with APPLE scores and survival at discharge was evaluated. Critically ill dogs had significantly greater positive fluid balance than the control group, and 8/16 dogs with substantial FO (12% or more) died. There was only a weak correlation between % FO and composite APPLE scores, which were based on clinical data that was not collected at a standardized time point and could have under- or overestimated the severity of illness.

More recently, a prospective observational study of dogs with acute kidney injury described the relationship between systemic hypertension and severity of kidney injury, and included observations about FO (40). In this report, FO was characterized as edema and was diagnosed based only on discretionary clinician assessment of acute weight gain, development of body cavity effusion, or physical examination findings. 22/52 dogs met the criteria for FO, and these dogs were significantly more likely to have hypertension and were more likely to die than dogs without.

Although these studies demonstrate potential harm of FO in clinical veterinary patients, a causal relationship between FO and outcome can't be demonstrated by a retrospective or observational study. Prospective interventional studies comparing the effect of standard (or liberal) fluid administration with fluid restriction on %FO and outcomes are needed to address this.

## Avoiding FO

Most reports of studies of FO have focused on the effect of fluid administration in the early hours to 1–2 days of treatment of life-threatening illness. Because of the growing evidence that FO is associated with worse outcomes in the critically ill, there has been considerable interest in validating non-invasive techniques to identify patients who respond to intravenous resuscitation fluid with an increase in cardiac output, and avoid (or at least limit) administration of fluids to those who do not. Those techniques are reviewed in detail by Boysen and Gommeren elsewhere in this issue; however, it is worth mentioning here that there are unresolved questions about the benefit of an increase in cardiac output immediately following a fluid bolus when that increase may be transient and yet result in longer-lasting edema. For example, Roger et al. demonstrated that of the septic patients who responded to a fluid bolus with an increase in cardiac stroke volume, half lost that benefit within 20 min (41). Most studies of techniques used to predict fluid responsiveness have not characterized patient responses beyond a few minutes, and it is quite possible that for many, a transient response to fluid infusion does not translate into a sustained benefit for circulation or outcome.

**Figure 1:**
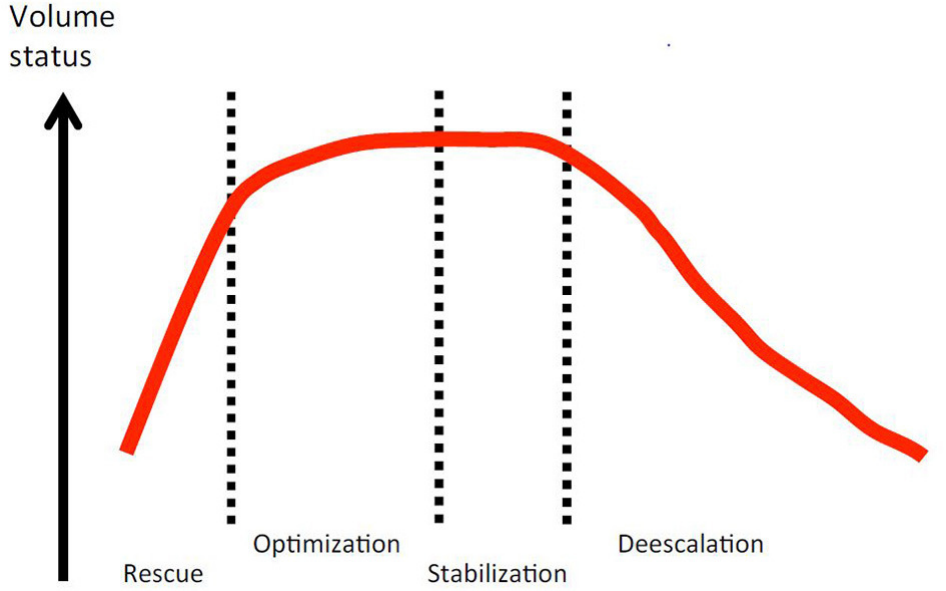
Conceptual relationship between patient volume status and the four phases of fluid therapy in critical illness. Open access image from the Acute Dialysis Quality Initiative 12, downloaded from https://www.adqi.org/Images on 3/15/2021.

Fluid therapy does not end with initial resuscitation and in fact often continues for days in the critically ill, in stages that have been characterized as rescue, optimization, stabilization, and de-escalation (Figure 1) (42). During initial rescue (resuscitation), most clinical decisions about fluid therapy are made within minutes and are based predominately on clinical signs. It is common to see unambiguous signs of positive hemodynamic responses to fluid administration in overtly hypovolemic patients, and physical examination alone or sometimes in combination with the ultrasound techniques noted above is often adequate to guide fluid dose and rate of administration and avoid hypervolemia. The optimization phase occurs over hours, or longer if the underlying disease is complex or progressive, for example sepsis or pancreatitis. During this phase fluid therapy may require ongoing administration of replacement type fluids that are titrated toward optimizing the circulation but avoiding an excess that will produce edema. It is at this stage that response to fluids may become much more nuanced and difficult to evaluate with physical examination alone, and using other techniques—for example, ultrasound and measurements related to oxygen delivery—become much more important. The stabilization phase occurs during recovery when the patient has become hemodynamically stable and fluid therapy shifts toward optimizing electrolyte balance, replacing normal and pathological ongoing losses, and the beginning of a negative fluid balance as the patient excretes the excess fluid administered during resuscitation and optimization. The de-escalation phase is characterized by a transition to self-sufficiency via oral intake and a negative fluid balance where FO had occurred.

The optimization and stabilization phases are times when development of FO due to inappropriate administration of excessive quantities of both water and sodium in “maintenance” fluids and fluid vehicles for drug administration is probably common. Routine practice in charting human fluid balance ignores the volume of fluid administered as a vehicle for drugs, and this “fluid creep” can create a large volume of unaccounted fluid administration that contributes to FO. In fact, maintenance fluid and fluid creep may account for the majority of parenteral water and sodium administration in human ICU patients (43). The common veterinary practice of using a high-sodium replacement fluid like lactated Ringer's or a proprietary equivalent, at volumes that are often much larger than required to replace normal ongoing losses in an immobilized critically ill dog or cat, likely contribute a great deal to the prevalence of FO. Maintenance requirements of water and electrolytes for dogs and cats may be very different from commercial fluid composition and commonly cited administration rates, as has been recently reviewed in detail elsewhere (44).

## Treatment of FO

The clearest indication for intervention in animals with FO is the presence of hypervolemia, which should be managed with sodium and water restriction, diuretics, and in selected life-threatening situations, hemofiltration when the potential for a diuretic response is impaired. The most obvious cases of hypervolemia include edematous animals with kidney injury causing oliguria or anuria, and animals with pulmonary edema secondary to heart failure. Physical examination alone is likely to correctly identify these and inform treatment. Diuretic treatment for left sided congestive heart failure usually consists of furosemide 1–2 mg/kg by intravenous or intramuscular injection, followed by an intravenous constant infusion (0.66 mg/kg/hour) for 6 h treatment blocks when venous catheterization can be obtained without compromising the patient (45). Animals with kidney injury and oliguria may have impaired delivery of the drug to site of action in the loop of Henle. Animals that do not respond to usual doses of furosemide (e.g., 2 mg/kg) may respond to high doses, e.g., 2 mg/kg repeated to as much as 8 mg/kg within 1–2 h, followed by a continuous infusion titrated to maintain the target rate of urine production.

Animals with other causes of FO and edema may be hyper-, normo-, or hypovolemic, and classification and treatment of these may be more difficult. For example, an animal with sepsis or pancreatitis may become edematous at a low MCFP because of changes in the microcirculation and interstitial matrix, and aggressive treatment with diuretics for imagined hypervolemia will compromise circulation and cause harm. These animals may develop relatively severe FO after even tiny increases in MCFP. If there is clinical evidence of impaired circulation that may respond to fluid therapy, benefit from resuscitation fluids should be predicted and monitored with adjunct assessment methods such as ultrasound measurement of dynamic variables, central or mixed venous oxygen saturation, and central venous or arterial blood pressure responses to incremental doses of fluids or drugs. Replacement fluids should be used only during the resuscitation and optimization phases, with an eye toward discontinuing them as soon as a transition to the stabilization phase is possible. Albumin solutions or plasma may be administered to animals with hypoalbuminemia that is sufficiently severe to contribute to edema formation. Although administration of albumin solutions instead of crystalloids to critically ill humans provides little improvement in most outcome measures, those patients generally require less total fluid for resuscitation/optimization and may have less tendency for FO (46–48). We have observed similar benefit from albumin or plasma administration to critically ill dogs in our ICU, reaching the stabilization phase with less tendency for FO. Although fresh frozen plasma is an inefficient way to provide albumin, some animals may benefit from coagulation factors or other plasma components. In our ICU, a common dosing strategy for animals in shock from sepsis and other causes of a systemic inflammatory response is to use plasma (and more recently, 5% canine albumin) as a component of resuscitation fluid therapy, then continue administration as a continuous rate infusion of ~20 mL/kg/day during the optimization phase. This fluid is always accounted for in total fluid balance calculations, and crystalloid administration is reduced by an equal amount.

Animals in septic shock are rarely resuscitated with fluid therapy alone. Removal of the underlying cause, for example infection source control, is a critical step to reversing pathology of the microcirculation and interstitial matrix to correct FO. Mechanical ventilation to reduce the work of breathing and decrease pulmonary shunt may be required to address an increase in extravascular lung water. Pharmacological management of the circulation, for example the administration of pressors to maintain MCFP, arterial pressure, and cardiac output, is routinely essential to correct hypotension restore adequate oxygen delivery and allow earlier transition to a stabilization phase with less fluid administration.

Once an animal with FO edema has reached the stabilization phase it may be much more tolerant of fluid restriction and graded diuretic therapy. In this stage, a low test dose (<1 mg/kg) of intravenous furosemide may be administered and the patient response evaluated. If urine output increases the patient is monitored for evidence of hypovolemia, and in its absence diuretic therapy may be continued with a goal of gradually reducing FO to <5% over 1–2 days. Fluid therapy at this stage is often provided partly or wholly via enteral nutrition, but if intravenous fluid is continued it should be restricted to water and electrolytes calibrated to meet metabolic need (44). By the time the animal reaches the de-escalation phase they are often capable of ambulation and spontaneous position change; this activity may hasten elimination of FO.

## Conclusion

Fluid overload is likely to be a detrimental and sometimes life-threating complication of disease that is much more likely to occur when fluid therapy is not carefully calibrated to maintain an adequate circulation without causing edema. It is clearly much better to avoid it in the first place rather than treat it after it appears. This is best accomplished by administering replacement fluids only to those patients capable of responding with an increase in oxygen delivery during the rescue and optimization phases of fluid therapy, and by using a protocol for maintenance fluid that is guided by careful consideration of the actual metabolic needs of the animal.

## Author Contributions

The author confirms being the sole contributor of this work and has approved it for publication.

## Conflict of Interest

The author declares that the research was conducted in the absence of any commercial or financial relationships that could be construed as a potential conflict of interest.

## Footnotes

^1^The original Holliday-Segar formula is 100 mL/kg/day for patients <10 kg, 1 liter + 50 mL/kg/day for patients 10–20 kg, and 1.5 L + 20 mL/kg/day for patients >20 kg. Thus, for a 22 kg patient the daily volume would be 1540 mL, or 70 mL/kg/day.
